# Matcha green tea targets the gut–liver axis to alleviate obesity and metabolic disorders induced by a high-fat diet

**DOI:** 10.3389/fnut.2022.931060

**Published:** 2022-08-01

**Authors:** Yuefei Wang, Yueer Yu, Lejia Ding, Ping Xu, Jihong Zhou

**Affiliations:** Tea Research Institute, Zhejiang University, Hangzhou, China

**Keywords:** obesity, tea polyphenols, metabolic regulation, bile acid, gut microbiota

## Abstract

Obesity induced by a high-fat diet (HFD) is an increasing global health problem, leading to many metabolic syndromes. As the emerging food additive rich in tea polyphenols, theanine, caffeine, and so on, matcha green tea has gained more and more popularity for its outstanding potential in ameliorating metabolic disorders. This study investigated the composition and antioxidant activity of matcha green tea and further explored its effects on gut–liver axis homeostasis in an HFD-induced obese mouse model. Male (7–8 weeks old) C57BL/6J mice were divided into four groups with the following dietary supplementation for 8 weeks: a normal chow diet (NCD), a normal chow diet+1.0% matcha (NCM), a high-fat diet (HFD), and a high-fat diet+1.0% matcha (HFM). The results demonstrated that matcha green tea ameliorated the development of obesity, lipid accumulation, and hepatic steatosis induced by HFD. Subsequently, dietary matcha supplementation restored the alterations in fecal bile acid profile and gut microbial composition. Meanwhile, the levels of mRNA expression in hepatocytes demonstrated that matcha intervention made significant regulatory on the multiple metabolic pathways of hosts involved in glucose, lipid, and bile acid metabolism. These findings present new evidence for matcha green tea as an effective nutritional strategy to mitigate obesity and relevant metabolic disorders through the gut–liver axis.

## Introduction

With the prevalence of the high-fat diet (HFD) and other unhealthy lifestyles, obesity has become a global public health concern in the past decades (1). As a chronic and progressive condition, obesity subsequently increases the risk of various metabolic disorders including hypertension osteoarthritis, type 2 diabetes, cardiovascular disease, and non-alcoholic fatty liver disease (NAFLD) (2–4). Therefore, there is an urgent need for strategies to prevent and treat obesity (5). The application of plant-derived functional food and food supplements in maintaining health and preventing diseases has attracted more and more attention in recent years (6–8).

Matcha green tea is a kind of powdered tea obtained from the leaves of the tea plant (*Camellia sinensis* (L.) Kuntze) grown under shading cultivation, with high amounts of tea polyphenols, amino acids, and chlorophyll (9, 10). It is widely used as a beverage or food ingredient due to its fresh taste and nice appearance color. Moreover, different from traditional green tea, both water-soluble and water-insoluble ingredients in matcha can be ingested, enhancing its health function potential. Some studies have reported that matcha has hypolipidemic, hypoglycemic, and anti-obesity effects (11, 12). Consistently, our previous study focused on liver function and found that matcha was effective in improving HFD-induced hepatitis and lipid metabolism disorders (13). However, the interactions between multiple organs and the clear mechanisms of matcha on HFD-induced obesity remained to be further explored.

Accumulating evidence showed that the gut microbiome plays a critical role in the development of obesity and relevant diseases, which is related to their cross talks with the liver, immune system, and central nervous system (14–17). The bidirectional communications between the gut microbiota and the host's metabolism carry out through multiple metabolites, including bile acids (BAs) and short-chain fatty acids (SCFAs) (18–21). Thus, targeting the variation in the composition of the microbiota and metabolites along the gut–liver axis could be a promising strategy for investigating the underlying mechanism of matcha in relieving obesity.

In this study, the composition and antioxidant activity of matcha green tea and its anti-obesity effects through gut–liver interactions were investigated. The relationship between bacterial composition and the BAs metabolic network in the gut was further investigated by 16S rRNA gene sequencing and targeted metabolomics study. Finally, we examined the expression levels of the relevant hepatic mRNA and performed a systematic correlation analysis, which might be helpful for our new understanding of the possible mechanisms underlying the anti-obesity effect of matcha.

## Materials and methods

### Chemical analysis of matcha samples

Matcha samples made from *Camellia sinensis* cv. Zhongcha 108, cv. Longjing 43, cv. Yingshuang, cv. Maolv, and cv. Quntizhong were obtained from Zhejiang Tea Group Co., Ltd. and stored at 4°C for further study.

The total phenolic content, soluble protein content, free amino acid content, total sugar content, contents of caffeine and tea catechins content, and water lixivium content were implemented according to our previous report (12). All chemical standards were of high-performance liquid chromatography grade.

### Antioxidant capacity assessment of matcha samples

The antioxidant capacities were measured by the indicators of the 2,2-diphenyl-1-pricylhydrazyl (DPPH) scavenging activity, the 2,2′-Azino-bis(3-ethylbenzothiazoline-6-sulfonic acid) diammonium salt (ABTS) radical scavenging activity, and ferric ion reducing antioxidant power (FRAP). The DPPH assay was carried out according to Sun et al. (22). The ABTS assay was carried out according to the method of Luo et al. (6) and the FRAP assay was carried out according to Zhang et al. (23). For each sample, the antioxidant index score and the antioxidant potency composite (APC) index were calculated. The matcha sample with the highest antioxidant ability was blended into the diet for further animal experiments.

### Animals and diets

All procedures of experimental animals were approved by the Committee on the Ethics of Animal Experiments of Zhejiang University (ethic approval code: ZJU20190065). The foodborne obesity mouse model was established according to our previous publication with slight modifications (24). Twenty male C57BL/6J mice (25 ± 2 g, 7–8 weeks old) were obtained from the Shanghai Laboratory Animal Center of the Chinese Academy of Sciences (Shanghai, China). All mice were bred with five animals per cage in the Laboratory Animal Center of Zhejiang University (Hangzhou, China) and maintained under controlled conditions (temperature: 22 ± 1°C, humidity: 55 ± 5%, conventional 12-h light/dark cyclic light environment). Food and water were obtained *ad libitum* during the experimental period. After a 1-week acclimation, the mice were randomly divided into four groups (*n* = 5 per group) and fed with the following supplement for 8 weeks: (1) NCD group: a normal chow diet (10% fat), (2) HFD group: a high-fat diet (45% fat), (3) NCM group: a normal diet blending with 1.0% matcha, and (4) HFM group: a high-fat diet blending with 1.0% matcha. The composition of experimental diets is shown in Supplementary Table S1. The normal chow diet (10% energy from fat, #D12450B) and high-fat diet (45% energy from fat, #D12451) for mice were supplied by the Research Diets, Inc. Co., Ltd. (New Brunswick, NJ, USA) (13). The body weight and food intake of each group of mice were measured weekly. The mice were sacrificed under isoflurane anesthesia at the end of the 8th week.

### Sample collection and preparation

Blood and tissue samples were collected from mice which were fasted for 12 h. Blood was collected into the 1.5 mL centrifuge tube and kept at room temperature for 2 h before centrifugation at 3,000 rpm, 4°C for 15 min. The serum was separated and stored at −80°C. The liver, epididymal white adipose tissue (eWAT), perirenal white adipose tissue (pWAT), subcutaneous white adipose tissue (sWAT), and interscapular brown adipose tissue (iBAT) were excised, weighed, and immediately frozen in liquid nitrogen for 15 min and which were finally stored at −80°C for subsequent experiments. After the intestinal samples were dissected, the intestinal contents were squeezed into a 5-mL sterile centrifuge tube by PBS and immediately frozen in liquid nitrogen before storing at −80°C until further analysis.

### Biochemical assays of the serum

The serum biochemical assays of serum glucose, total cholesterol (TC), triacylglycerol (TG), high-density lipoprotein (HDL), low-density lipoprotein (LDL), and the serum enzymes alanine aminotransferase (ALT) and aspartate aminotransferase (AST) were determined by an automatic biochemical analyzer (TBA-40FR, Toshiba Medical, Tokyo, Japan).

### Histopathological examination of adipose tissue and liver

The adipose tissue samples were collected and fixed in 4% paraformaldehyde at 4°C for 24 h before being embedded in paraffin. The 5-μm sections cut from the blocks were stained with hematoxylin and eosin (H&E) for general morphological observations. The liver samples were stained with oil red O according to the kit instructions (Solarbio, Beijing, China) before being visualized by a microscope.

### Gut microbiota analysis

The gut microbial genomic DNA was extracted from the colonic contents using TruSeq® DNA PCR-Free Sample Preparation Kit. The V3-V4 region of the 16S rRNA gene was amplified by PCR using the primers 341F (5-CCTAYGGGRBGCASCAG-3) and 806R (5-GGACTACNNGGGTATCTAAT-3) and then fully sequenced on the NovaSeq PE250 platform (Illumina, USA) from Wuhan Metware Biotechnology Co, Ltd (Wuhan, China). After being quality-filtered, the paired-end reads were turned into the tags which were assigned to operational taxonomic units (OTUs) with a cut-off value of 97%. Taxonomic assignment was based on the SILVA_138 database. The Alpha-diversity (Chao1 index) and Beta-diversity (PCoA index) analyses were calculated with Qiime 2 software and displayed with R software. Stool microbial characterization was subjected to linear discriminant analysis (LDA) effect size (LEfSe). The Spearman's correlation analysis between lipid-related traits and the key intestinal microbial phylotypes was calculated by psych package and visualized by heatmap package.

### Targeted profiling of fecal bile acids

Fecal bile acid contents were detected by Wuhan Metware Biotechnology Co, Ltd (Wuhan, China) based on the AB Sciex QTRAP 6500 LC-MS/MS platform. The original UPLC-MS/MS data were processed by MultiQuant software (Version 3.0.3) for peak detection, alignment, and standardization. Principal component analysis (PCA) was performed by statistics function prcomp within R. The data were unit variance scaled before unsupervised PCA. Orthogonal partial least squares discriminate analysis (OPLS-DA) generated using the R package MetaboAnalystR was used for the statistical analysis to determine the metabolic changes between the groups. Variable importance in the projection (VIP) was calculated in the OPLS-DA model to select the potential biomarkers and the *p*-value was determined by the two-tailed *t*-test (VIP > 1.0 and *p* < 0.05).

### Quantitative reverse transcription PCR (qRT-PCR)

The hepatic RNA was extracted using the Trizol reagent according to the manufacturer's protocol and was reversely transcribed using a cDNA reverse transcription kit (Invitrogen, Carlsbad, CA, USA). Synthesized cDNA was amplified using the SYBR Green PCR Master Mix (Applied Biosystems, Foster City, CA, USA) on the LightCycler480 real-time system (Roche, Switzerland). The mRNA level of each gene was normalized to the geometric mean of the β-Actin mRNA level and expressed as values of relative expression compared to that of the NCD group. The sequences of the primers used in this study are listed in Supplementary Table S2.

### Statistical analysis

The statistical analysis and graphic illustration were performed using GraphPad Prism version 9.0 (GraphPad Software Inc., San Diego, CA, USA) and SPSS Statistics version 26.0 (IBM Corporation., Armonk, NY, USA). Results were expressed as mean ± standard error of the mean (SEM). The statistical significance was indicated by the one-way analysis of variance (ANOVA) followed by Tukey's multiple comparison test. The *p*-value less than 0.05 (*p* < 0.05) was rated as statistically significant and the statistical differences were indicated with superscript letters.

## Results

### Bioactive compounds and antioxidant activity of the matcha samples

Components were the basis of matcha quality characteristics and biochemical functions, which differed from cultivars (25). The major compounds of five matcha samples were determined and shown in Table 1. HPLC analysis was used to determine the amount of catechins monomers and caffeine. Among the five matcha samples in this study, cv. Maolv was the richest in free amino acid and protein, while cv. Longjing 43 had the highest soluble sugar content and tea polyphenols (TP) content. The content of EGCG was significantly higher than that of other catechins and there were a lot of ester catechins in all matcha samples. The results showed that the content of EGCG was significantly higher than that of other catechins and there were a lot of ester catechins in all matcha samples.

**Table 1:** Chemical analysis of the matcha samples.

**Cultivars**	**Zhongcha 108**	**Longjing 43**	**Quntizhong**	**Yingshuang**	**Maolv**
Water content	3.28 ± 0.02^e^	3.66 ± 0.08^d^	3.97 ± 0.04^c^	4.12 ± 0.08^b^	6.20 ± 0.05^a^
Water extracts content	40.86 ± 0.72^b^	42.20 ± 0.26^a^	39.15 ± 0.35^c^	40.46 ± 0.23^b^	37.15 ± 0.37^d^
Free amino acid	5.27 ± 0.13^b^	4.98 ± 0.23^c^	5.39 ± 0.08^b^	4.98 ± 0.16^c^	5.90 ± 0.09^a^
Protein	2.56 ± 0.18^b^	2.66 ± 0.35^ab^	2.67 ± 0.32^ab^	2.69 ± 0.21^ab^	3.11 ± 0.22^a^
Soluble Sugar	6.65 ± 0.04^b^	7.14 ± 0.03^a^	6.66 ± 0.13^b^	6.54 ± 0.09^b^	6.60 ± 0.18^b^
Tea polyphenols	11.97 ± 0.03^bc^	12.43 ± 0.24^a^	11.73 ± 0.16^c^	11.03 ± 0.17^d^	12.16 ± 0.24^ab^
Caffeine	2.25 ± 0.24^c^	2.46 ± 0.25^bc^	2.90 ± 0.34^ab^	3.12 ± 0.38^a^	3.26 ± 0.21^a^
GC	0.20 ± 0.06^a^	0.25 ± 0.01^a^	0.22 ± 0.02^a^	0.11 ± 0.04^b^	0.20 ± 0.02^a^
EGC	1.40 ± 0.14^b^	1.63 ± 0.21^ab^	1.49 ± 0.14^ab^	0.65 ± 0.10^c^	1.80 ± 0.13^a^
C	0.05 ± 0.01^b^	0.06 ± 0.01^b^	0.06 ± 0.01^b^	0.02 ± 0.01^c^	0.08 ± 0.01^a^
EC	0.34 ± 0.04^b^	0.41 ± 0.06^ab^	0.37 ± 0.03^b^	0.21 ± 0.04^c^	0.44 ± 0.03^a^
EGCG	5.00 ± 0.30^b^	4.88 ± 0.51^b^	5.81 ± 0.32^ab^	6.38 ± 0.82^a^	6.47 ± 0.55^a^
GCG	0.11 ± 0.02^ab^	0.09 ± 0.01^b^	0.11 ± 0.01^ab^	0.12 ± 0.02^a^	0.13 ± 0.01^a^
ECG	0.81 ± 0.07^b^	0.86 ± 0.10^b^	1.03 ± 0.07^a^	0.81 ± 0.12^b^	1.17 ± 0.09^a^
CG	0.04 ± 0.01^a^	0.03 ± 0.01^b^	0.04 ± 0.01^ab^	0.02 ± 0.01^c^	0.04 ± 0.00^ab^
GA	0.03 ± 0.00^cd^	0.03 ± 0.00^d^	0.04 ± 0.00^bc^	0.06 ± 0.01^a^	0.05 ± 0.00^b^

Data are expressed as means ± SEM (n = 3). Means with different letters (a-e) were considered significantly different at p < 0.05 according to Tukey's test. GC, (+)-gallocatechin; EGC, (-)-epigallocatechin; C, (+)-catechin; EC, (-)-epicatechin; EGCG, (-)-epigallocatechin gallate; GCG, (+)-gallocatechin gallate; ECG, (-)-epicatechin gallate; CG, (-)-epigallocatechin gallate; GA, gallic acid.

Epigallocatechin gallate (EGCG), epigallocatechin (EGC), epicatechin gallate (ECG), and epicatechin were the main active compounds in catechins. As having the ability to neutralize free radicals and boost the detoxification activity of enzymes, catechins were a kind of natural antioxidants in tea and matcha was their best-condensed source. Free radicals and antioxidants played key roles in the regulation of metabolism. Compounds with high biological activity, like flavonoids and flavons from buckwheat (26), vitamin E from olive oil or sunflower seed oil (27), polysaccharides from guava leaves (6), and alginate oligosaccharides (7) diminished negative influence on metabolisms of a high-fat diet by antioxidant effects. Thus, the antioxidant abilities of five samples were measured comprehensively from three perspectives to select the best variety to make chow feed, which laid a foundation for subsequent animal experiments.

The antioxidant activity of the matcha samples were shown in Table 2. The results demonstrated that cv. Zhongcha 108 and cv. Longjing 43 showed good antioxidant capacity when the antioxidant activity was evaluated by the DPPH method. Cv. Longjing 43 showed the highest antioxidant capacity in the ABTS method. The determination results of the FRAP method were different from those of the other two methods. Therefore, the APC index was adopted in this study to comprehensively evaluate the antioxidant capacity of five matcha samples from different methods. For each sample, antioxidant index score = [(sample score/best score) × 100]. The APC index was calculated as the average of the antioxidant index scores of the referred three methods. Sample made from cv. Longjing 43 ranking first among all five cultivars was used for subsequent animal experiments.

**Table 2:** Antioxidant activity of the matcha samples.

**Cultivars**	**DPPH**	**ABTS**	**FRAP**	**APC index**	**Rank**
Zhongcha 108	26.63 ± 0.10^a^	19.94 ± 0.02^b^	41.46 ± 0.38^c^	98.87%	2
Longjing 43	26.45 ± 0.11^b^	20.03 ± 0.04^a^	41.89 ± 0.22^b^	99.14%	1
Quntizhong	25.88 ± 0.12^d^	19.46 ± 0.04^c^	41.76 ± 0.15^bc^	97.38%	5
Yingshuang	26.21 ± 0.10^c^	19.12 ± 0.02^d^	42.70 ± 0.16^a^	97.95%	4
Maolv	26.14 ± 0.10^c^	19.48 ± 0.05^c^	42.11 ± 0.18^b^	98.01%	3

Data are expressed as means ± SEM (n = 3). Means with different letters (a-d) were considered significantly different at p < 0.05 according to Tukey's test.

### Matcha supplementation ameliorated the obesity and reduced fat accumulation caused by high-fat diets

**Figure 1:**
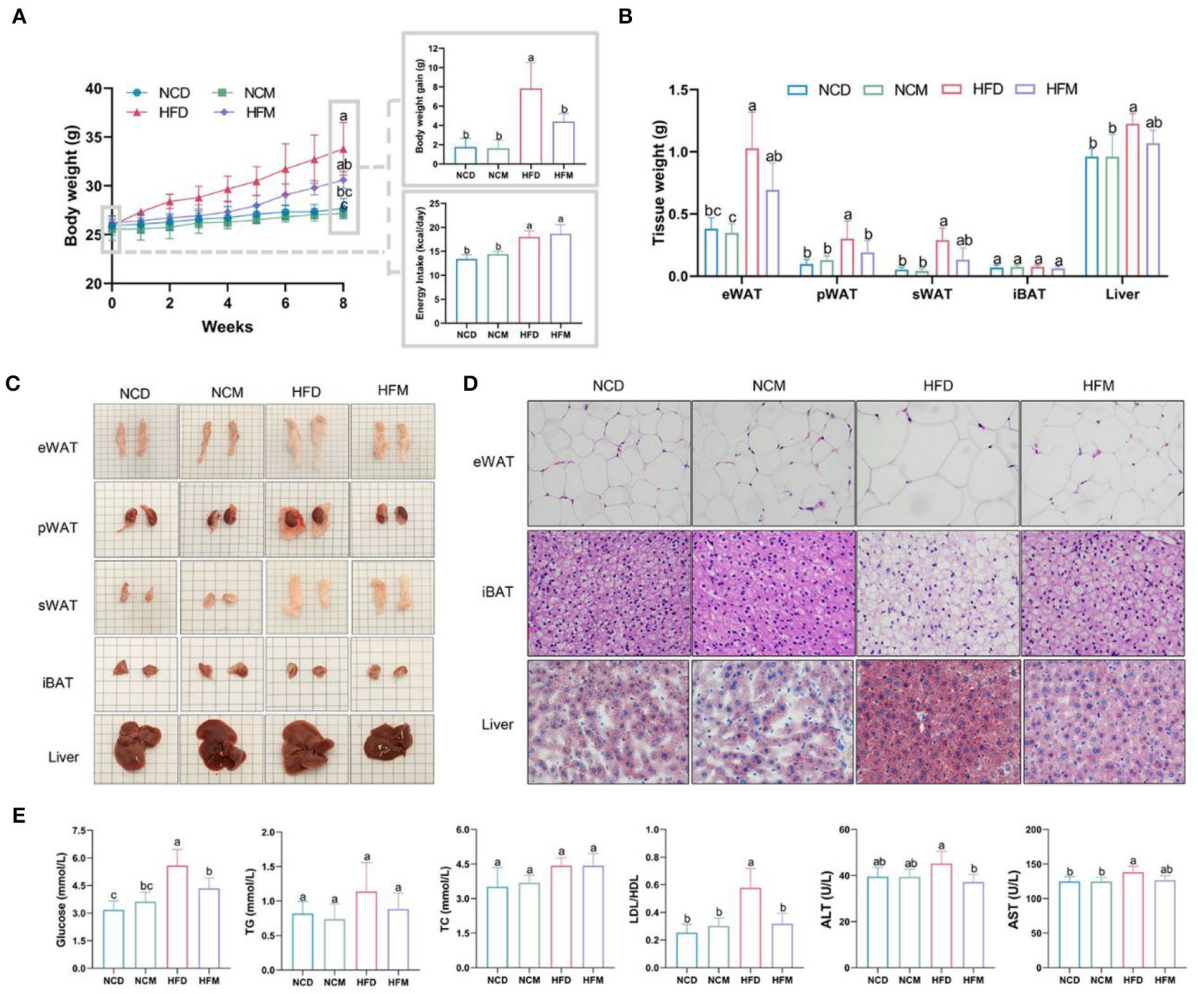
Effect of matcha supplementation on high-fat diet-induced obesity and serum biochemical parameters. **(A)** Body weight evolution and energy intake. **(B)** Tissue weight. **(C)** Representative images of the adipose tissues and livers. **(D)** Hematoxylin and eosin (H&E) staining of eWAT, iBAT, and liver samples. **(E)** Serum biochemical parameters. Data are expressed as means ± SEM (*n* = 5). Means with different letters (a-c) were considered significantly different at *p* < 0.05 according to Tukey's test. NCD, mice on a normal chow diet; HFD, mice on a high-fat diet; NCM, mice on a normal chow diet with 1.0% matcha; HFM, mice on a high-fat diet with 1.0% matcha.

Effects of matcha on the high-fat-diet-induced obesity indexes were shown in Figure 1. After 8 weeks of experimental treatment, the high-fat diet induced a notable increase in body weight gain and fat accumulation in pWAT and sWAT, as well as lipid deposition in epididymal adipocytes and liver. Supplementation with matcha mitigated the effect of HFD in the weights of body gain, liver, pWAT, and sWAT without affecting the energy intake (Figures 1A–C). Histological analysis showed excessive lipid accumulation and infiltration in the HFD group, which were decreased with the matcha supplementation (Figure 1D). The morphological results of the liver tissues revealed that the high-fat diet induced significant hepatic steatosis, lipid droplets accumulation, and cell rupture, which was improved by matcha supplementation (Figure 1D). No significant difference in related parameters between the NCD group and the NCM group indicated that matcha was not harmful to the normal physiological conditions of mice.

As shown in Figure 1E, the serum levels of glucose, TG, TC, LDL/HDL ratio, ALT, and AST in the HFD mice were significantly higher than in the NCD mice. The ALT and AST index were considered key biochemical indicators of hepatic injury degree. Comprehensively considering the morphological results and the significant decrease of AST and ALT indexes in the HFM group compared with the HFD group suggested that matcha can help the liver maintain stability to prevent the metabolism disorder induced by the high-fat diet.

### Effects of matcha on the levels of fecal BAs

**Figure 2:**
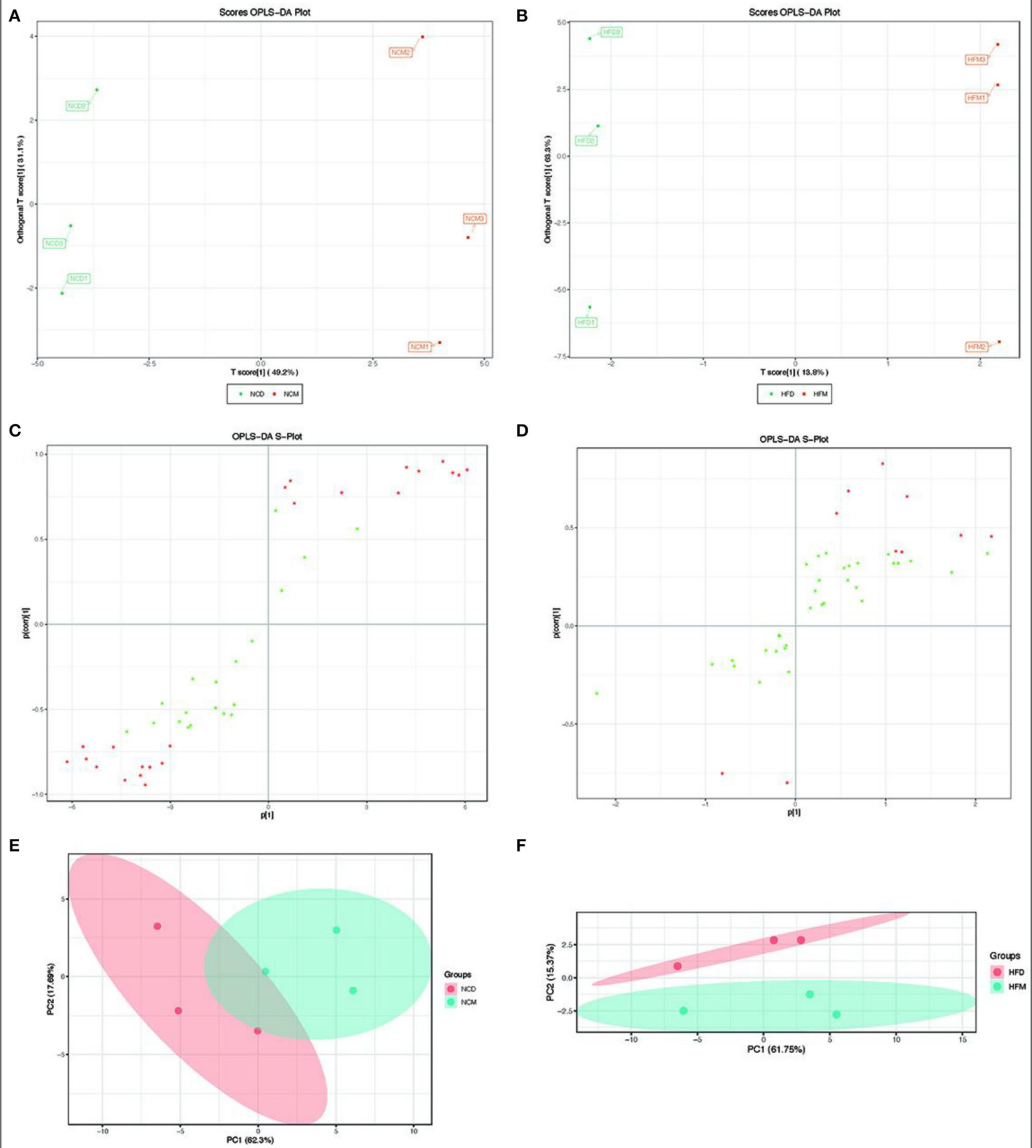
Effects of matcha supplementation on the composition and proportion of fecal bile acids. **(A)** OPLS-DA score plot of NCD and NCM groups. **(B)** OPLS-DA score plot of HFD and HFM groups. **(C)** S loading plot based on OPLS-DA Analysis model of NCD and NCM groups. **(D)** S loading plot based on OPLS-DA Analysis model of HFD and HFM groups. **(E)** PCA plot of NCD and NCM groups. **(F)** PCA plot of HFD and HFM groups. NCD, mice on a normal chow diet; HFD, mice on a high-fat diet; NCM, mice on a normal chow diet with 1.0% matcha; HFM, mice on a high-fat diet with 1.0% matcha.

**Figure 3:**
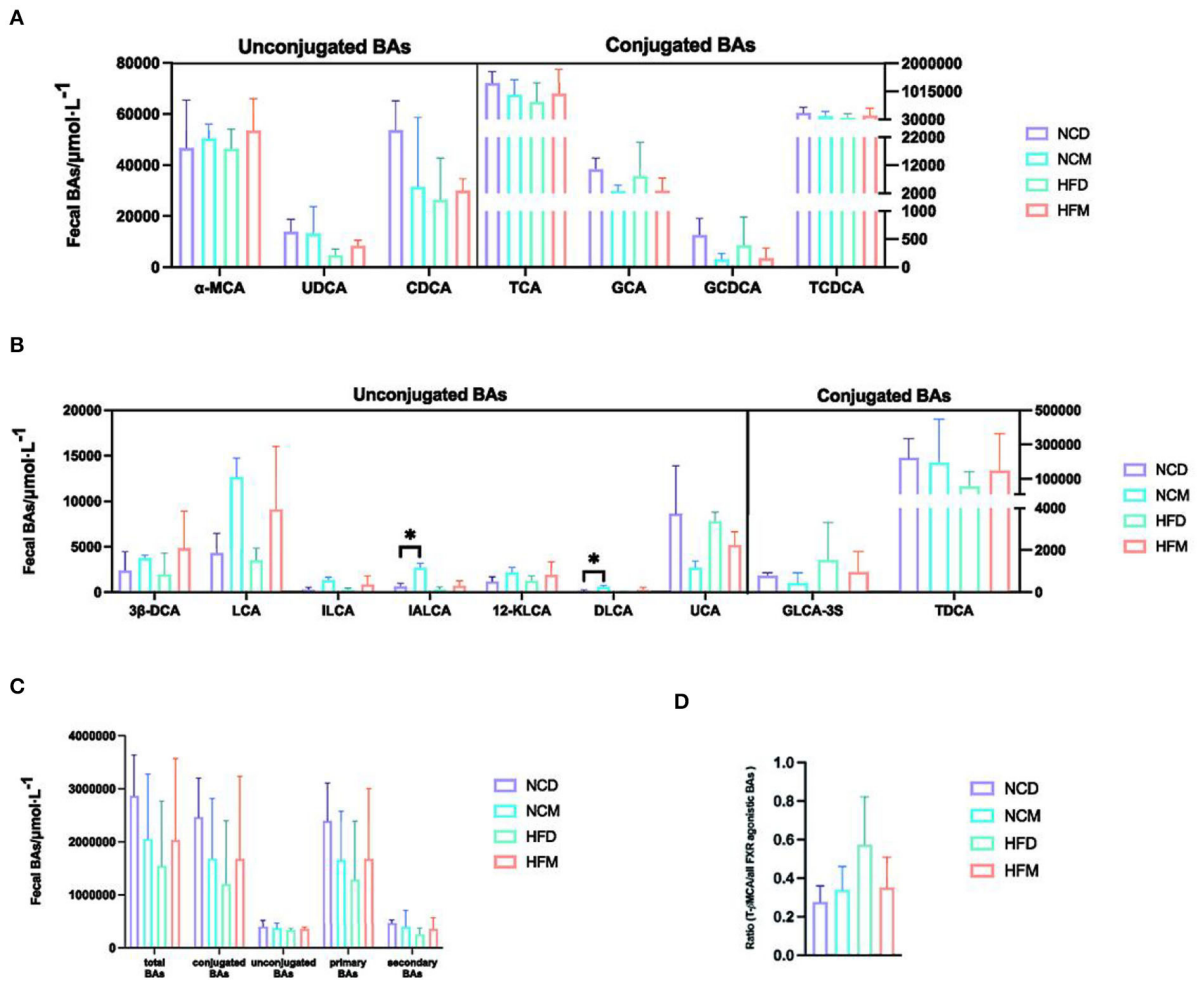
Effects of matcha supplementation on the fecal bile acids (BAs) levels. **(A)** Levels of primary BAs. **(B)** Levels of secondary BAs. **(C)** Levels of summarized BAs. **(D)** The ratio between T-βMCA (FXR antagonist) and the pool of FXR agonistic BAs (TCA, TCDCA, TDCA, TLCA, CA, CDCA, DCA, LCA). Data are expressed as means ± SEM (*n* = 5). Asterisk was considered significantly different at *p* < 0.05 according to Tukey's test. NCD, mice on a normal chow diet; HFD, mice on a high-fat diet; NCM, mice on a normal chow diet with 1.0% matcha; HFM, mice on a high-fat diet with 1.0% matcha.

The effects of matcha on fecal BA levels were illustrated in Figures 2, 3. Score plots of PCA exhibited distinguished classification in group NCD with NCM (Figure 2E), and HFD with HFM (Figure 2F). Based on the OPLS-DA model, the score plots showed that there was an obvious separation in NCD with NCM (Figure 2A), and HFD with HFM (Figure 2B). As shown in Figures 2C,D, the bile acids in the loading plot away from the coordinate center were considered potential biomarkers responsible for group separation (28). Although not all groups showed significant differences in the changes of BAs, BAs presented a good tendency in the way matcha improves obesity. As shown in Figures 3A,B, α-muricholic acid (α-MCA), 3β-deoxycholic acid (3β-DCA), lithocholic acid (LCA), isolithocholic acid (ILCA), isoallolithocholic acid (IALCA), 12-ketolithocholic acid (12-kLCA), and dehydrolithocholic acid (DLCA) were upregulated in matcha supplied groups while glycocholic acid (GCA), ursocholic acid (UCA), and glycolithocholic acid-3-sulfate (GLCA-3S) were notably downregulated. Matcha intervention showed different effects on a normal diet and a high-fat diet in some types of BAs as chenodeoxycholic acid (CDCA), taurocholic acid (TCA), taurochenodeoxycholic acid (TDCA), and summarized BAs demonstrated in Figure 3C, which suggested that matcha may play an important role in regulating bile acid homeostasis. The ratio between tauro-β-muricholic acid (T-βMCA) (Figure 3D), the proportion of a potent FXR antagonist, and a pool of known agonistic BAs (TCA, TCDCA, TDCA, TLCA, CA, CDCA, DCA, and LCA) are also shown to stabilize under matcha supplementation.

### Matcha modulated intestinal microbial populations

**Figure 4:**
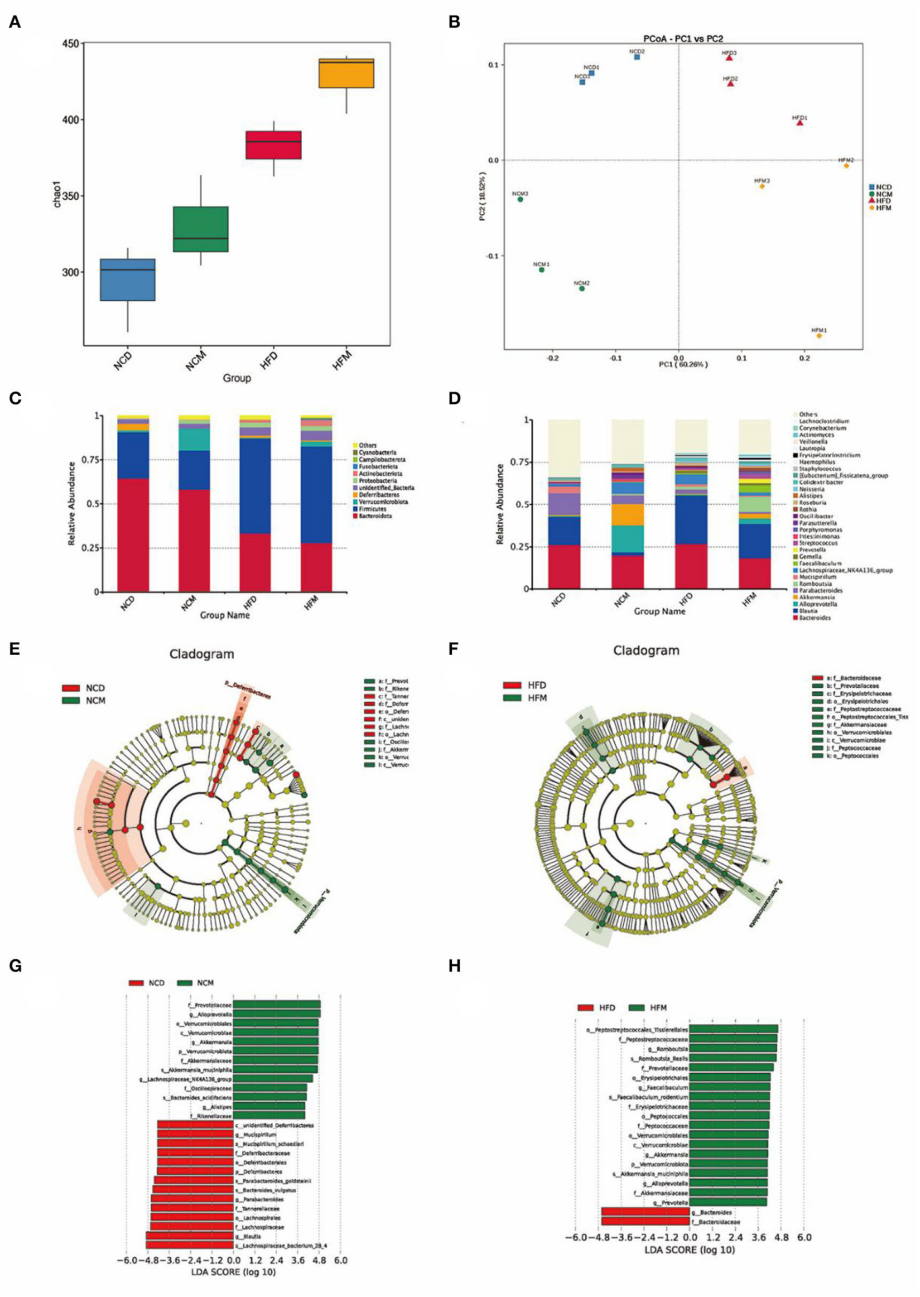
Effects of matcha supplementation on gut microbiota. **(A)** Alpha diversity of the gut microbiome community of four groups. The diversity was assessed within the QIIME2 pipeline based on the Chao1 index. **(B)** Principal coordinates analysis (PCoA) of the gut microbiome community structure. The community clustering is based on Bray–Curtis dissimilarities (Weighted UniFrac). **(C)** Relative abundance of gut microbiota at the phylum level. **(D)** Relative abundance of intestinal microbiota at the genus level. LEfSe analysis of intestinal microbiota composition based on relative abundances of 16S rRNA. LEfSe cladogram **(E,F)** representing different abundant taxa and LDA scores **(G,H)** as calculated by LEfSe analysis. Only taxa with LDA scores of more than 3 were presented. NCD, mice on a normal chow diet; HFD, mice on a high-fat diet; NCM, mice on a normal chow diet with 1.0% matcha; HFM, mice on a high-fat diet with 1.0% matcha.

The intestinal microbiota structures of four groups were analyzed using 16S rRNA gene amplicon sequencing. A total of 1,094,746 raw sequences were obtained. After sequence processing, 660,094 effective tags of 16S rRNA (accounting for 60.3% of the raw sequences) with an average length of 416 bp were obtained. Sequences were classified into species-level OTUs at 97% similarity for each sample. A total of 806 OTUs were obtained from all samples, ranging from 382 to 582 OTUs per sample. These OTUs were then categorized into 15 phyla, 25 classes, 59 orders, 92 families, and 176 genera by comparison to a standard database (Silva 138 database). Alpha-diversity and Beta-diversity were calculated to reveal the effect of high-fat diet treatment and matcha invention on the intestinal microbiota. Differences in Chao 1 index (Figure 4A) and significant separation of intestinal microbiota in four groups in the PCoA plot (Figure 4B) both indicate that matcha supplementation could modulate the composition of gut microbiota in mice.

To evaluate specific changes in the gut microbiota, the relative abundance of four groups (Figures 4C,D) was analyzed at different taxonomic levels. The gut microbiota was dominated (≥ 0.5% of all sequences across all samples) by the phyla Bacteroidota and Firmicutes in all groups. At the phylum level, matcha supplementation significantly increased phylum Verrucomicrobiota while decreasing Deferribacteres. Compared with the NCD group (42.0%), Firmicutes/Bacteroidota ratio in the NCM group (38.8%) was decreased significantly. As shown in Figures 4E–H, matcha supplementation affected the gut microbiota by enriching the genus *Alloprevotella*, which was the producer of short-chain fatty acids (SCFAs) and the abundances of which were negatively correlated with non-alcoholic fatty liver. Furthermore, *Akkermansia muciniphila*, as the emerging probiotics, was increased in both the NCM group and HFM group, while *Mucispirillum schaedleri*, which were considered to be related to nonalcoholic fatty liver and nonalcoholic steatohepatitis (NASH), was observed to be suppressed in NCM group.

### Effect of matcha on mRNA expression levels of hepatic genes involved in lipid metabolism

**Figure 5:**
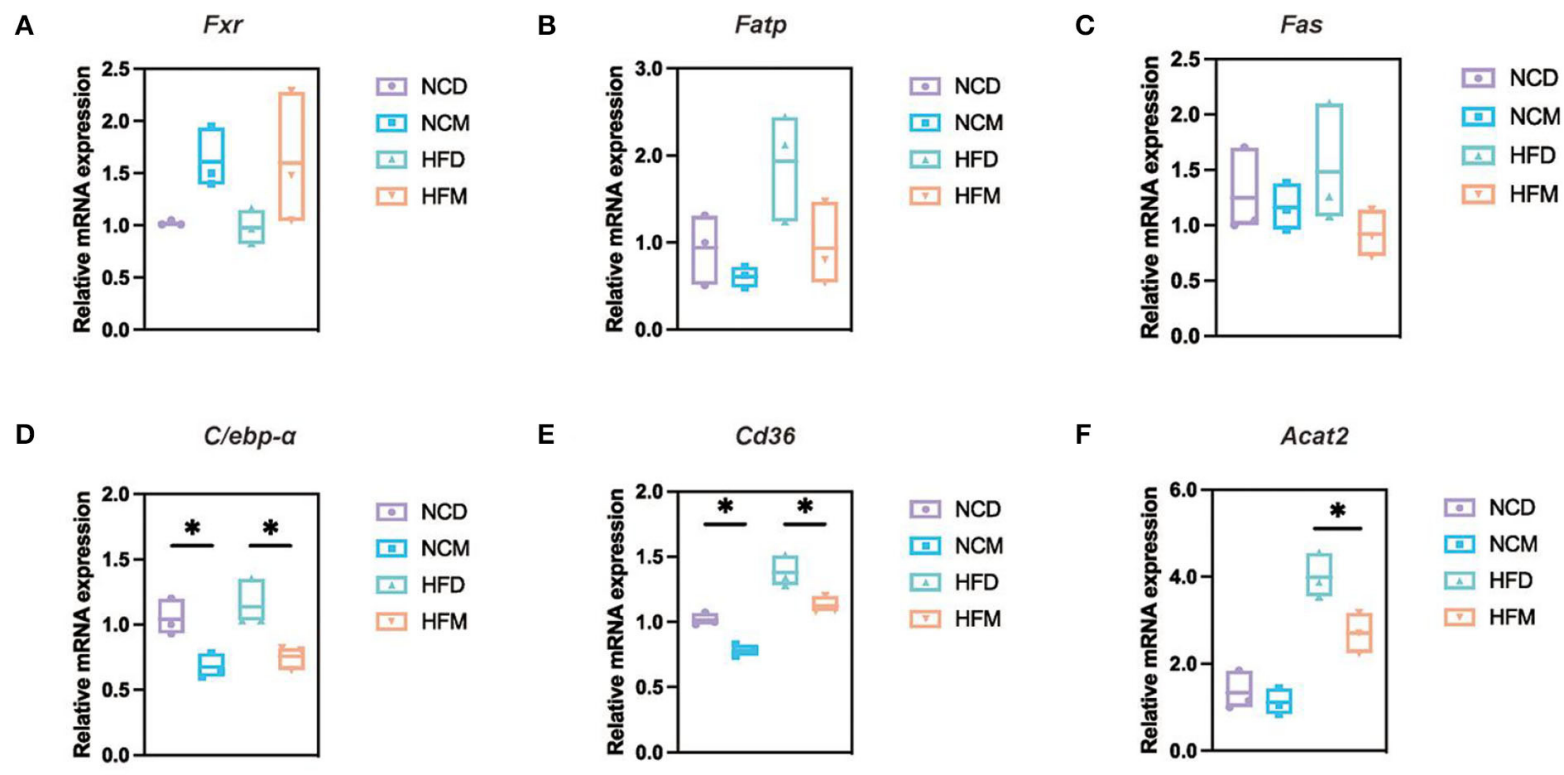
Effects of matcha supplementation on liver mRNA expression levels related to lipid metabolism and bile acid homeostasis. **(A)** The expression levels of *Fxr*. **(B)** The expression levels of *Fatp*. **(C)** The expression levels of *Fas*. **(D)** The expression levels of *C/ebp-*α. **(E)** The expression levels of *Cd36*. **(F)** The expression levels of *Acat2*. Data are expressed as means ± SEM (*n* = 3). Asterisk was considered significantly different at *p* < 0.05 according to Tukey's test. NCD, mice on a normal chow diet; HFD, mice on a high-fat diet; NCM, mice on a normal chow diet with 1.0% matcha; HFM, mice on a high-fat diet with 1.0% matcha.

To evaluate the mechanisms underlying the amelioration effect of matcha supplementation on NAFLD, the levels of hepatic mRNA expressions involved in lipid metabolism and BA homeostasis were determined by qRT-PCR (Figure 5). It was shown that matcha supplement led to similar variation trends in the levels of mRNA expression in both normal diet groups and high-fat diet groups, indicating that matcha supplementation had consistent regulating mechanisms on dyslipidemia and BA homeostasis. Supplementation of matcha upregulated the expression levels of farnesoid X receptor (*Fxr*), while markedly downregulated the levels of fatty acid transporter protein (*Fatp*), fatty acid synthase (*Fas*), CCAAT/enhancer-binding proteins-alpha (*C/ebp-*α), cluster of differentiation 36 (*Cd36*), and acetyl-CoA acetyltransferase 2 (*Acat2*).

### Correlations of the key microbial phylotypes with lipid metabolic parameters

**Figure 6:**
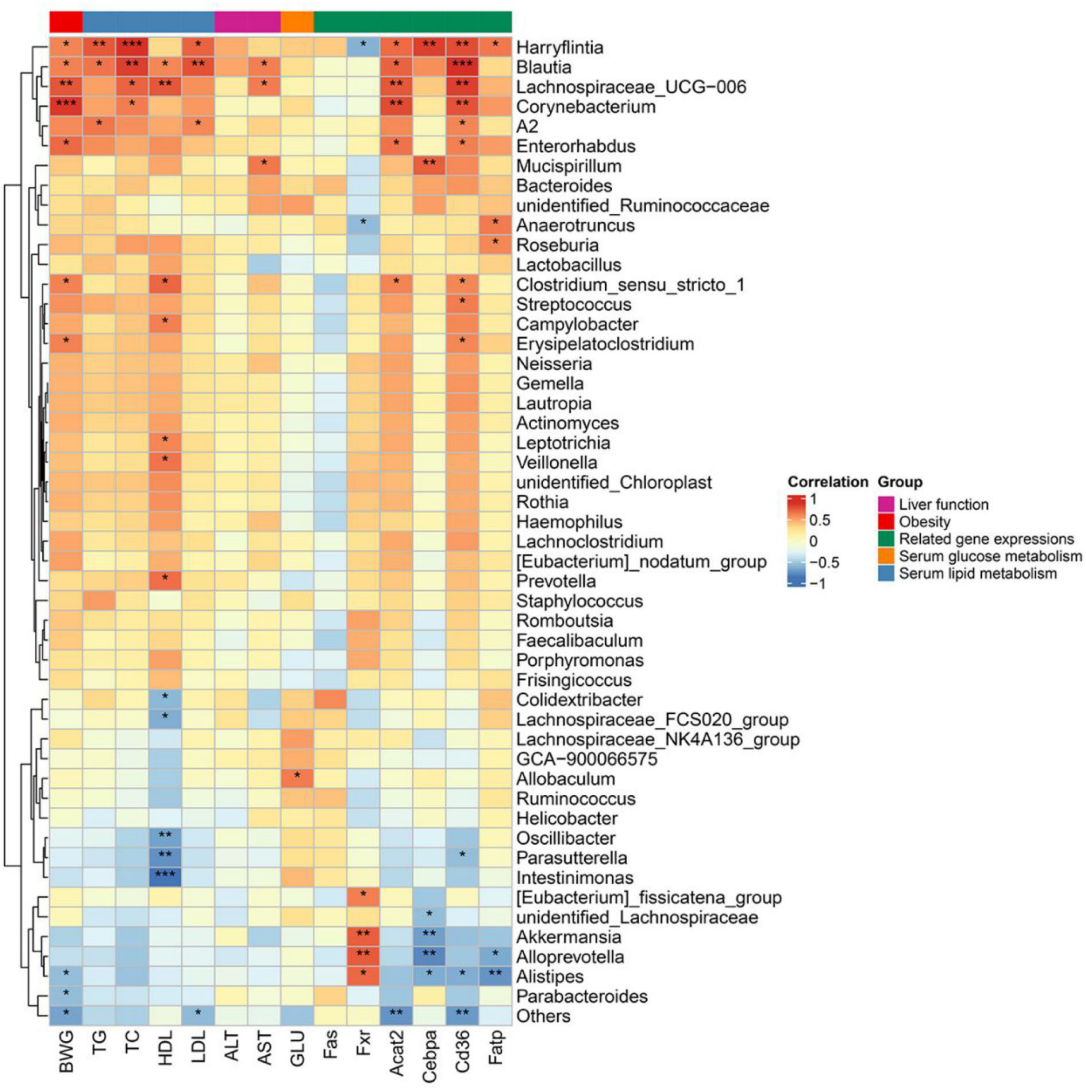
Spearman's correlation analysis of the top 50 abundance intestinal microbiota at the genus level with obesity traits and metabolic parameters. The degree of red indicates that the relationship between them tends to be positively correlated. In contrast, the blue degree indicates that the relationship between them tends to be negatively correlated. Asterisk denotes *p* < 0.05, double asterisk denotes *p* < 0.01, triple asterisk denotes *p* < 0.005.

To further explore the relationship between the altered gut microbiota, obesity traits, and metabolic parameters, Spearman's correlation analysis was performed as shown in Figure 6. The abundant microbiota in the intestine showed different correlations with obesity traits and metabolic parameters such as body weight gain, serum parameters, and lipid metabolism-related gene expressions. The results suggested that the matcha treatment could regulate gut microbiota in mice, thus improving the metabolism disability induced by the high-fat diet.

## Discussion

As a trendy drink and dietary supplement, matcha, an ultra-fined green tea powder, has drawn intensive interest in research because of its potential intervention ability in obesity and relevant metabolic diseases (12, 13, 24). Being consistent with the observations in previous literature, here we found that matcha can efficiently inhibit fat accumulation as well as ameliorate dyslipidemia and dysglycemia caused by obesity. However, the mechanism of matcha in improving lipid metabolism remains to be further studied.

Gut microbiota has been characterized to have related underlying mechanisms in obesity for it plays a key role in energy homeostasis, immunity, and blood circulation (29–31). More and more evidence proved that compounds with high biological activity, such as polyphenols, can help modulate the gut microbiota dysbiosis and intervene the obesity (32–34). Chemical analysis showed that matcha supplied in this study was rich in EGCG, an antioxidant with high biological activity. The results of high-throughput sequencing (HTS) revealed that matcha shaped the intestinal microbiota of mice by affecting both its diversity and composition. Matcha supplementation increased the relevant abundance of key intestinal genera such as SCFAs producers (*Faecalibaculum, Alloprevotella*). The Genus *Romboutsia* that was more abundant in the HFM group vs. the HFD group might play a part in maintaining host health and was found to be negatively associated with body weight, fasting serum glucose, and insulin in previous research (35, 36). *Akkermansia muciniphila* enriched in both normal diet and high-fat diet after matcha intervention, which was consistent with the previous *in vivo* research using antioxidant compounds (37). As a mucin-degrading bacterium, *Akkermansia muciniphila* was found to be an emerging probiotic in preventing obesity and relevant metabolic diseases (38, 39). The abundance of which was positively associated with fatty acid oxidation and the browning of white adipocytes, while negatively correlated with inflammation and metabolic syndrome makers (40). Although specific mechanisms underlying the beneficial impact of *A. muciniphila* on health have not been fully illustrated, previous studies revealed that *A. muciniphila* could modulate mucus thickness as well as gut barrier integrity, and thereby contributes to metabolism normalization (41). The effect of *A. muciniphila* on the host depends on interactions with other gut microorganisms and suggests remaining cautious in using *A. muciniphila* as a probiotic treatment (42). It was also found that the antioxidant may protect *A. muciniphila*, an obligate anaerobe, from free oxygen radicals (43). A safer approach, such as polyphenol-rich extracts, to thrive *A. muciniphila* abundance in the gut thus alleviating lipid metabolism disorders is more reliable (42). However, Matcha intervention significantly altered the gut microbiota, but the relationship between matcha intervention and lipid metabolism regulation needs to be further clarified.

BAs, as the important signal metabolites and regulators of bidirectional communication between the intestinal microbiome and the host, performed key pathophysiological functions along the gut–liver axis through a variety of host receptors (44–47). Moreover, BA metabolism was considered to be linked with lipid metabolism (48). Effects of matcha intervention on the fecal BAs presented a steady tendency. Namely, matcha supplementation alleviated the changes in BAs induced by the high-fat diet and adjusted the levels of BAs to similar standards of mice fed with different diets. It is generally considered that the liver is the most important organ for modulating the biosynthesis and metabolism of cholesterol and BAs (49). In this study, mRNA expression levels of hepatic genes involved in BAs homeostasis and lipid metabolism were also investigated. The upregulation of genes *Fas, Acat2*, and *Fatp* suggested that hepatic lipogenesis and fatty acid transport in lipid accumulation were enhanced in HFD group mice, which were consistent with the changes in biochemical parameters of the liver. The expression of *C/ebp-*α, the negative regulator of insulin gene transcription, was downregulated after matcha intervention. *C/ebp-*α was reported to be the key regulator of adipogenesis for modulating the expression of its target genes, such as ap2 (activating protein) directly implicated in lipogenic pathways in 3T3-L1 adipocytes, during the early to middle stage of differentiation (50, 51). The decrease in expression of *C/ebp-*α may be related to the anesis of hepatocyte lipid droplets accumulation and lipid deposition in matcha-supplied mice. As liver steatosis is a key marker of NAFLD, it is a confirmed strategy to inhibit the progression of NAFLD in its early stages by reducing the TG accumulation through regulating the related genes of free fatty acids (FFAs) uptake and lipid synthesis pathways in the liver (52, 53). Matcha intervention inhibited the upregulated expression of *Cd36*, consequently decreasing the uptake and transmembrane transfer of FFAs (54). *Cd36* is also responsible for promoting the formation of high TG in the blood. The decrease of serum TG may be due to the suppression of the *Cd36* expression. Therefore, the possible mechanism of matcha intervention in regulating metabolism disorders induced by a high-fat diet may be achieved by maintaining BAs homeostasis, modulating relevant hepatocyte gene expressions, and accelerating the conversion of FFAs and BAs. The western blot test should be taken in the future for further exploration and verifying the results at the protein level.

The gut microbiota played a pivotal role in multiple metabolic pathways in the host (55, 56). The correlation analysis of the gut microbiota and metabolites highlights the relevance of the gut microbiota with obesity-associated metabolic parameters. *Blautia*, from the family Lachnospiraceae, was reported to play a key role in the production of acetic and butyric acids (57) and was found to have a positive correlation with most obesity traits and metabolic-related parameters in this study. Previous literature found that *Blautia* was positively associated with most of the lipid metabolism-related parameters (49) and showed a significant increase in type 2 diabetic rats induced by HFD (58). *Akkermansia, Alistipes*, and *Alloprevotella* showed a negative correlation with body weight gain and liver-related gene expression like *C/ebp-*α, the negative regulator of insulin gene transcription (51). Among which, genus *Alistipes* appeared to be significantly negatively related to the expression of *C/ebp-*α, *Cd36*, and *Fatp* in Spearman's correlation analysis. *Alistipes* were generally considered as the producers of SCFAs and promoting probiotics for they may provide energy to the host and affect the production of antimicrobial peptides (59–61). Based on the correlation analysis, it can be reasonably proposed that the possible underlying regulatory mechanism of matcha supplementation in preventing obesity and relevant metabolism dysfunction might be through the regulation of gut microorganisms, thus maintaining bile acid homeostasis and lipid metabolism balance. Key floras like *Alistipes, Blautia*, and *Akkermansia* might play crucial roles in the prevention effect of matcha against HFD-induced obesity. However, the specific relationship between specific gut microbiota and lipid metabolic parameters remains to be further verified by fecal microbiota transplantation (FMT).

## Conclusion

**Figure 7:**
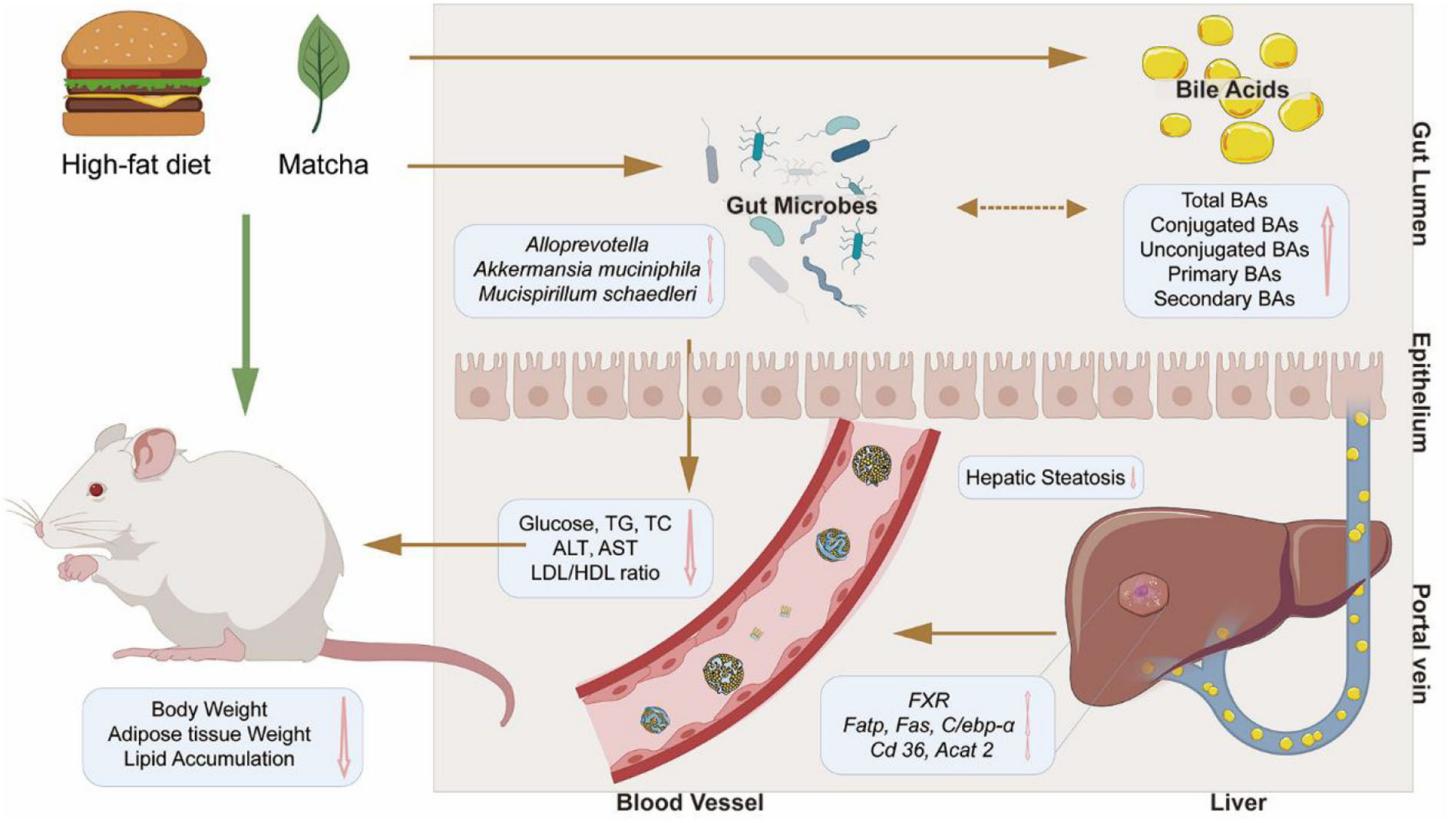
Schematic diagram showing the possible mechanisms of matcha green tea preventing high-fat diet-induced obesity through the gut–liver axis.

As shown in Figure 7, the improvement in biochemical parameters, the diversity and compositions of key flora, and the proportion of fecal BAs levels illustrate that the related gut–liver axis is the potential regulatory target of matcha in improving lipid accumulation and metabolic disorders. These effects may be due to the downregulation of *Fatp, Fas, C/ebp-*α*, Cd36*, and *Acat2* levels and the upregulation of *Fxr* level in hepatocytes. In particular, matcha treatment enriched SCFAs producers such as *Faecalibaculum, Alloprevotella*, and potential probiotics such as *Akkermansia muciniphila* and provide useful information for excavating functional probiotics with hypolipidemic activity. Taken together, this study provides new evidence for matcha green tea as a promising food supplementation against HFD-induced obesity.

## Data availability statement

The datasets presented in this study can be found in online repositories. The names of the repository/repositories and accession number(s) can be found below: https://www.ncbi.nlm.nih.gov/, PRJNA833235.

## Ethics statement

The animal study was reviewed and approved by the Committee on the Ethics of Animal Experiments of Zhejiang University (ethic approval code: ZJU20190065).

## Author contributions

YW and JZ conceived the study. YW, LD, and YY performed the experiments and analyzed the data. YW, YY, and JZ contributed to the discussion of the work and assisted in drafting the manuscript. JZ and PX revised the manuscript. All authors have read and agreed to the final version of the manuscript.

## Funding

This work was supported by a key joint grant for regional innovation from the National Natural Science Foundation of China (Grant Number U19A2034).

## Conflict of interest

The authors declare that the research was conducted in the absence of any commercial or financial relationships that could be construed as a potential conflict of interest.

## Publisher's note

All claims expressed in this article are solely those of the authors and do not necessarily represent those of their affiliated organizations, or those of the publisher, the editors and the reviewers. Any product that may be evaluated in this article, or claim that may be made by its manufacturer, is not guaranteed or endorsed by the publisher.
