# Supplementary material for: Matcha green tea targets the gut–liver axis to alleviate obesity and metabolic disorders induced by a high-fat diet
Source: Front Nutr. 2022 Aug 1;9:931060. doi: 10.3389/fnut.2022.931060 (PMC9376390; doi:10.3389/fnut.2022.931060)
Supplement: Supplementary file 1 [file Table_1.DOCX]

Supplementary Material

**Table S1.** Compositions of animal diets

| **Ingredient** | **Normal chow diet (#D12450B)** | | **High-fat diet (#D12451)** | |
| --- | --- | --- | --- | --- |
|  | **g** | **kcal** | **g** | **kcal** |
| Casein, 80 Mesh | 200 | 800 | 200 | 800 |
| L-Cystine | 3 | 12 | 3 | 12 |
| Corn Starch | 315 | 1260 | 72.8 | 291.2 |
| Maltodextrin 10 | 35 | 140 | 100 | 400 |
| Sucrose | 350 | 1400 | 172.8 | 691.2 |
| Cellulose, BW200 | 50 | 0 | 50 | 0 |
| Soybean Oil | 25 | 225 | 25 | 225 |
| Lard | 20 | 180 | 177.5 | 1598 |
| Mineral Mix S10026 | 10 | 0 | 10 | 0 |
| DiCalcium Phosphate | 13 | 0 | 13 | 0 |
| Calcium Carbonate | 5.5 | 0 | 5.5 | 0 |
| Potassium Citrate, 1 H_2_0 | 16.5 | 0 | 16.5 | 0 |
| Vitamin Mix V1001 | 10 | 40 | 10 | 40 |
| Choline Bitartrate | 2 | 0 | 2 | 0 |
| FD&C Yellow Dye #5 | 0.05 | 0 | 0 | 0 |
| FD&C Red Dye #40 | 0 | 0 | 0.05 | 0 |
| Total | 1055.05 | 4057 | 858.15 | 4057 |

**Table S2.** Primer sequences for the targeted mouse genes

| **Name** | **Reverse (5′ to 3′)** | **Forward (3′ to 5′)** |
| --- | --- | --- |
| *Fxr* | GGCACTCCCATTTACAGGCTA | TGAACTTGAGGAAACGGGACA |
| *Fatp* | TGCTCTATGACTGCCTGCCA | GCAGGTAGCGGCAGATTTCA |
| *Fas* | GGCGGGTTCGTGAAACTGAT | GCTCAGCCTAGTTTTCAGGTTGG |
| *C/ebp-α* | AAGAAGTCGGTGGACAAGAACAG | CGTTGCGTTGTTTGGCTTTATC |
| *Cd36* | CTGTGGCTAAATGAGACTGGGA | ACCACTCCAATCCCAAGTAAGG |
| *Acat2* | CACAGAACAGGGCAGAGCAT | CCATCGTTCATTCCTGATGCG |
| *Gapdh* | CCTCGTCCCGTAGACAAAATG | TGAGGTCAATGAAGGGGTCGT |

*Fxr*, farnesoid X receptor; *Fatp*, fatty acid transporter protein; *Fas*, fatty acid synthase; *C/ebp-α*, CCAAT/enhancer-binding proteins-alpha; *Cd36*, cluster of differentiation 36; *Acat2*, acetyl-CoA acetyltransferase 2; *Gapdh*, glyceraldehyde 3-phosphate dehydrogenase.

**Table S3.** List of the abbreviation used trough the paper.

| **Abbreviation** | **Complete spelling** |
| --- | --- |
| HFD | high-fat diet |
| HFM | high-fat diet+1.0% matcha |
| NCD | normal chow diet |
| NCM | normal chow diet+1.0% matcha |
| NAFLD | non-alcoholic fatty liver disease |
| SCFAs | short-chain fatty acids |
| BAs | bile acids |
| FRAP | Ferric ion reducing antioxidant power |
| APC | antioxidant potency composite |
| eWAT | epididymal white adipose tissue |
| pWAT | perirenal white adipose tissue |
| sWAT | subcutaneous white adipose tissue |
| BAT | brown adipose tissue |
| H&E | hematoxylin and eosin |
| TC | total cholesterol |
| TG | triacylglycerol |
| HDL | high-density lipoprotein |
| LDL | low-density lipoprotein |
| ALT | glutamic pyruvic transaminase |
| AST | glutamic oxaloacetic transaminase |
| EGCG | Epigallocatechin gallate |
| EGC | epigallocatechin |
| ECG | epicatechin gallate |
| NASH | nonalcoholic steatohepatitis |
| *Fxr* | farnesoid X receptor |
| *Fatp* | fatty acid transporter protein |
| *Fas* | fatty acid synthase |
| *C/ebp-α* | CCAAT/enhancer-binding proteins-alpha |
| *Cd36* | cluster of differentiation 36 |
| *Acat2* | acetyl-CoA acetyltransferase 2 |
| *Gapdh* | glyceraldehyde 3-phosphate dehydrogenase. |
| HTS | high-throughput sequencing |
| α-MCA | α-muricholic acid |
| 3β-DCA | 3β-deoxycholic acid |
| LCA | lithocholic acid |
| ILCA | isolithocholic acid |
| IALCA | isoallolithocholic acid |
| DLCA | dehydrolithocholic acid |
| 12-kLCA | 12-ketolithocholic acid |
| UCA | ursocholic acid |
| GCA | glycocholic acid |
| GLCA-3S | glycolithocholic acid-3-sulfate |
| CDCA | chenodeoxycholic acid |
| TCA | taurocholic acid |
| TDCA | taurochenodeoxycholic acid |
| T-βMCA | Tauro-β-muricholic acid |
